# LACTB suppresses liver cancer progression through regulation of ferroptosis

**DOI:** 10.1016/j.redox.2024.103270

**Published:** 2024-07-18

**Authors:** Kaixuan Zeng, Na Huang, Nanbin Liu, Xi Deng, Yanhua Mu, Xurui Zhang, Jian Zhang, Chongyu Zhang, Yong Li, Zongfang Li

**Affiliations:** a Department of Geriatric Hepatobiliary, Pancreatic and Spleen Surgery, The Second Affiliated Hospital of Xi'an Jiaotong University, Xi'an, 710004, China; b National-Local Joint Engineering Research Center of Biodiagnosis & Biotherapy, The Second Affiliated Hospital of Xi'an Jiaotong University, Xi'an, 710004, China; c Tumor and Immunology Center of Precision Medicine Institute, The Second Affiliated Hospital of Xi'an Jiaotong University, Xi'an, 710004, China; d Shaanxi International Cooperation Base for Inflammation and Immunity, The Second Affiliated Hospital of Xi'an Jiaotong University, Xi'an, 710004, China

**Keywords:** LACTB, Ferroptosis, Liver cancer, Lenvatinib

## Abstract

Ferroptosis, driven by iron-dependent phospholipid peroxidation, is emerging as an intrinsic cancer defense mechanism. However, the regulatory networks involved in ferroptosis remain largely unknown. Here, we found that serine beta-lactamase-like protein (LACTB) inhibits liver cancer progression by regulating ferroptosis. LACTB is downregulated in liver cancer, and the ectopic expression of LACTB markedly inhibits cell viability, colony formation, and tumour growth. LACTB knockout exerts the opposite effects. Further investigation revealed that LACTB blocks HSPA8 transcription in a p53-dependent manner, resulting in the elevation of NCOA4-mediated ferritinophagy and inhibition of SLC7A11/GSH/GPX4 signalling, thereby triggering ferroptosis and suppressing liver cancer progression. Liver cancer cells with an endogenous mutation of p53 binding site in the HSPA8 promoter exhibited increased resistance to ferroptosis inducers, and the ferroptosis-promoting effect of LACTB was significantly weakened in these mutant cells. Importantly, LACTB is identified as a downstream target of lenvatinib, and adeno-associated virus-mediated overexpression and knockdown of LACTB notably enhance and attenuate the anti-tumour efficacy of lenvatinib *in vivo*, respectively. Taken together, our study reveals a novel action of LACTB and provides potential therapeutic strategies for enhancing the efficacy of lenvatinib in liver cancer.

## 1 Introduction

Liver cancer is the sixth most commonly diagnosed cancer and the third leading cause of cancer-related death worldwide [1], with an estimated incidence of >1 million cases by 2025 [2]. Owing to the lack of specific clinical manifestations in the early stages, most patients are diagnosed with advanced liver cancer [3]. Only 5%–15 % of patients are eligible for surgical removal, and chemotherapy and immunotherapy are the best treatment options for the remaining patients [4,5]. Lenvatinib, an oral small-molecule tyrosine kinase inhibitor, represents an alternative to sorafenib for first-line targeted treatment of unresectable liver cancer [6]. Lenvatinib exerts antitumour effects by targeting multiple molecules, such as VEGFR and FGFR family members [7]. Although lenvatinib has demonstrated excellent efficacy in the clinical treatment of liver cancer, drug resistance is inevitable in targeted cancer therapies [8]. Therefore, the elucidation of the key molecular events driving liver cancer progression will provide new targets for clinical treatment and may lead to the development of combination therapies to improve lenvatinib efficacy.

Ferroptosis is a novel form of regulated cell death that differs from apoptosis, necrosis, pyroptosis, and autophagy, which are iron-dependent forms of cell death caused by reactive oxygen species-mediated accumulation of lipid peroxides [9,10]. Iron homeostasis in the body is tightly regulated. Intracellular iron is stored in ferritin, and the selective cargo receptor, NCOA4, can directly bind to ferritin and promote ferritin autophagic-lysosomal degradation and concomitant iron release, a process known as ferritinophagy [11]. NCOA4-mediated ferritinophagy is required to maintain intracellular and systemic iron homeostasis, excessive free iron triggers the Fenton reaction and facilitates the production of lipid peroxidation to initiate ferroptosis [12]. Multiple antioxidant systems exist in cells that resist ferroptosis, with the SLC7A11 (also commonly known as xCT)/glutathione (GSH)/GPX4 axis being the most extensively studied [13]. Extracellular cystine is transported into cells by SLC7A11 on the cell membrane, followed by its reduction to cysteine for the synthesis of GSH, then, GPX4 uses GSH as a cofactor to detoxify lipid peroxides to lipid alcohols, thereby suppressing ferroptosis [14]. The SLC7A11/GSH/GPX4 antioxidant axis is frequently dysregulated and is critical for tumour development [15]. Tumour cells grow uncontrollably and develop drug resistance by avoiding ferroptosis via multiple mechanisms; thus, targeted interventions for ferroptosis are essential for cancer treatment and prevention, as well as for improving the efficacy of clinical chemotherapy, radiotherapy, and immunotherapy [16,17].

Serine beta-lactamase-like protein (LACTB), an active-site serine protease, has recently become a topic of interest because of its potential role in lipid metabolism and tumorigenesis [18]. Its role as a tumour suppressor or oncogene depends on the context. Low LACTB expression has been observed in glioblastoma [19], melanoma [20], ovarian cancer [21], breast cancer [22,23]and colorectal cancer [24]. Overexpression of LACTB inhibits cancer cell proliferation, migration, and invasion. In contrast, LACTB functions as an oncogene in pancreatic adenocarcinoma [25] and nasopharyngeal carcinoma [26] by regulating different signalling pathways. Notably, one study proposed that LACTB is downregulated in liver cancer, which is associated with a poor prognosis [27]; however, the mechanism by which LACTB acts has not been elucidated. In addition, LACTB has been reported to promote erastin-induced ferroptosis and mitochondrial dysfunction in bladder cancer cells [28], but it is unclear whether LACTB is an intrinsic trigger of ferroptosis. In this study, we tested the expression levels of LACTB in liver cancer and investigated its biological functions and underlying mechanisms. We found that LACTB suppressed liver cancer progression by promoting ferroptosis via modulation of the p53/HSPA8 axis. Moreover, LACTB increased the sensitivity of liver cancer cells to lenvatinib.

## 2 Materials and methods

### 2.1 Chemicals

Erastin (#S7242), RSL3 (#S8155), ferrostatin-1 (#S7243), necrostatin-1 (#S8037) and Lenvatinib (#S1164) were purchased from Selleck Chemicals (TX, USA). Liproxstatin-1 (#HY-12726), deferoxamine (#HY-B1625) and *N*-acetyl-cysteine (#HY-B0215) were purchased from MedChem Express (NJ, USA). *Z*-VAD-FMK (#V116) was purchased from Sigma-Aldrich.

### 2.2 Tissues and cell lines

Ten pairs of freshly frozen liver cancer and adjacent normal tissues were surgically resected, collected, and immediately transferred to liquid nitrogen. Additionally, 12 normal and 31 paraffin-embedded liver cancer tissue samples were used to detect LACTB protein expression. A tissue microarray containing 71 liver cancer samples was used to test the correlations between LACTB, p53, and HSPA8. Clinical specimens were used with the informed consent of all patients, and the study was approved by the Biomedical Ethics Committee of the Xi'an Jiaotong University Health Science Center. HepG2 (#HB-8065, ATCC, VA, USA), SK-HEP-1 (#HTB-52, ATCC), Huh7 (#JCRB0403, JCRB Cell Bank, Tokyo, Japan), and Hep3B (#HB-8064, ATCC) cells were cultured in DMEM supplemented with 10–20 % foetal bovine serum (FBS) (20 % FBS for HepG2 cells). All the cells were verified using Short Tandem Repeat profiling.

### 2.3 Western blot

Total protein was collected using RIPA lysis buffer containing 0.5 M Tris-HCl, 1.5 M NaCl, 10 % NP-40, 10 mM EDTA and 2.5 % deoxycholic acid. Then, 10–30 μg protein was electrophoresed and transferred onto PVDF membrane. After incubation with the corresponding primary and secondary antibodies, protein signalling was visualised using the SuperSignal West Atto reagent (Thermo Fisher Scientific, CA, USA). The antibodies used in this study are as follows: *anti*-LACTB (#18195-1-AP, Proteintech, IL, USA), anti-4HNE (#MAB3249-SP, BD Systems, MN, USA), *anti*-HSPA8 (#M01024-1, BOSTER, CA, USA), *anti*-SLC7A11 (#ab300667, Abcam, Cambridge, UK), *anti*-GPX4 (#MAB5457, BD Systems), *anti*-NCOA4 (#A5695, ABclonal, MA, USA), *anti*-FTH1 (#A19544, ABclonal), *anti*-p53 (#sc-126, Santa Cruz Biotechnology, Texas, USA), *anti*-ubiquitin (#10201-2-AP, Proteintech) and *anti*-GAPDH (#60004-1-Ig, Proteintech).

### 2.4 Immunohistochemistry (IHC) staining

Paraffin-embedded tissues were dewaxed and dehydrated, followed by heat-induced antigen retrieval using a sodium citrate buffer (pH 6.0). The sections were then incubated with *anti*-LACTB (#18195-1-AP, Proteintech), *anti*-p53 (wild-type) (#MABE339, Sigma-Aldrich), and *anti*-HSPA8 (#M01024-1, BOSTER) primary antibodies and a DAKO peroxidase-labelled secondary antibody. IHC staining was conducted using the H-score method as previously described [29].

### 2.5 Vectors, oligonucleotides and transfection

The full-length coding sequence of human LACTB was cloned into the pHBLV-CMV-MCS-ZsGreen-Puro lentiviral vector (HANBIO, Shanghai, China), followed by infection of HepG2 and SK-HEP-1 cells. Cells stably expressing LACTB were obtained after screening with puromycin. The pcDNA3.1 (+) vector (Addgene, MA, USA) was used to express HSPA8 and p53. The verified siRNAs targeting HSPA8 (5′-GCAACTGTTGAAGATGAGAAA-3′) and p53 (5′-CGGCGCACAGAGGAAGAGAAT-3′) were synthesised by Sangon (Shanghai, China). Cell transfection was performed using Lipofectamine 3000 (Invitrogen, Carlsbad, CA, USA) or LipoFiter reagent (HANBIO) according to the manufacturer's instructions.

### 2.6 CRISPR/Cas9 gene editing technology

To generate LACTB^−/−^ cell lines, two sgRNAs (sg-LACTB#1:5′-CAGGTCGCGGCTGCTCTCGA-3′; sg-LACTB#2:5′-TAGGATGAGGTGGGCGCACC-3′) were synthesised and inserted into lenti-CRISPR v2 plasmid (#52961, Addgene). After co-transfection of HEK293T cells with psPAX2 and pMD2. G vectors, the viral supernatant was collected for infection of HepG2 and SK-HEP-1 cells. After screening with puromycin, LACTB knockout cells were obtained and verified by Sanger sequencing and Western blot. To generate cells with endogenous mutation of p53 binding site in HSPA8 promoter, gRNA (5′-ACCCTTTACTGGCTTTGACA-3′) was inserted into pSpCas9(BB)-2 A-Puro vector (#62988, Addgene), followed by electroporation with the synthesised ssODN into SK-HEP-1 cells using Lonza Amaxa 4D-Nucleofector. A limited dilution method was used to obtain single clones from the 96-well plates. Homozygous mutant cell lines were verified using Sanger sequencing and used in subsequent experiments.

### 2.7 Cell counting kit (CCK)-8 and colony formation assays

Cell viability was assessed using the CCK-8 assay. In brief, cells were plated in 96-well plates and treated with 10 μL CCK-8 solution (#HY-K0301, MedChemExpress) at 37 °C for 2 h. The absorbance at 450 nm was measured using a microplate reader. For the colony formation assay, 500 cells were plated in 6-well plates and cultured for 10 days. Colonies on the plates were fixed with absolute methanol for 15 min and stained with 0.1 % crystal violet for 10 min.

### 2.8 Animal study

Mice were subcutaneously injected with SK-HEP-1 cells and grown for four weeks under specific pathogen-free conditions, and tumour volume was recorded weekly. To assess the *in vivo* effect of LACTB on lenvatinib, when the volume of subcutaneous tumour reached about 0.05–0.1 cm^3^, mice were treated with lenvatinib (4 mg/kg, oral gavage) for five days per week, or in combination with one intratumoral injection of 1 × 10^12^ vg/mL adeno-associated virus expressing LACTB or sh-LACTB. Four weeks later, the mice were sacrificed, and the tumour weight was recorded. All mice survived at the end of the treatment and no mice exhibited severe loss of body weight (>15 %) or evidence of infections or wounds. The patient-derived xenograft (PDX) model was established as previously described [30], when the volume of subcutaneous tumour reached about 0.05–0.1 cm^3^, mice were intratumorally injected with adeno-associated virus expressing vector or LACTB. The animal study was approved by the Biomedical Ethics Committee of Xi'an Jiaotong University Health Science Center.

### 2.9 Quantitative reverse-transcription PCR (qRT-PCR)

Total RNA was extracted using TRIzol solution (Invitrogen), followed by reverse transcription and quantification using PrimeScript RT Master Mix (Takara Bio, Otsu, Japan) and SYBR Premix Ex Taq Kit (Takara Bio), according to the manufacturer's instructions. The following are primer sequences used in this study.

PTGS2: 5′-TGAATGGGGTGATGAGCAGT-3′ (forward), 5′-GGGATGCCAGTGATAGAGGG-3′ (reverse);
HSPA8: 5′-GCCTACCTTGGGAAGACTGT-3′ (forward), 5′-GCACGTTTCTTTCTGCTCCA-3′ (reverse);
GAPDH: 5′-GGATTTGGTCGTATTGGGCG-3′ (forward), 5′-ATCGCCCCACTTGATTTTGG-3′ (reverse). Ct values were normalised to those of GAPDH.

### 2.10 RNA sequencing

LACTB-expressing and control SK-HEP-1 cells were treated with TRIzol reagent (Invitrogen) and immediately transferred to liquid nitrogen. The following steps were performed by Majorbio Bio-Pharm Technology Co., Ltd. (Shanghai, China). Raw data were analysed using the Majorbio Cloud Platform (www.majorbio.com).

### 2.11 Detection of Fe^2+^, lipid peroxidation, MDA, GSH levels and cell death

Intracellular Fe^2+^ was detected by FerroOrange staining. In brief, cells were washed three times with hank's balanced salt solution (HBSS) and incubated with 1 μM FerroOrange working solution (#36104, Cell Signalling Technology, MA, USA) for 30 min. The signal intensity was observed using a confocal microscope. Lipid peroxidation was tested by liperfluo staining, cells were incubated with 10 μM liperfluo reagent (#L248, Dojindo, Kumamoto, Japan) for 30 min at 37 °C. The cells were then collected for flow cytometry analysis with a 488 nm channel. The levels of MDA and GSH were evaluated using the Lipid Peroxidation MDA Assay kit (#S0131, Beyotime, Beijing, China) and the GSH & GSSG Assay kit (#S0053, Beyotime), respectively, according to the manufacturer's instructions. Cell death was assessed using propidium iodide (#HY-D0815, MedChemExpress) staining, followed by flow cytometry analysis of a minimum of 10,000 cells.

### 2.12 Ubiquitination assay

The protocol for *in vivo* ubiquitylation assay was described in our previous study [30]. Cells were treated with 5 μM MG132 (#M7449, Sigma-Aldrich) for 6 h prior to collection. The antibody used for immunoprecipitation was *anti*-p53 (#sc-126; Santa Cruz Biotechnology).

### 2.13 Luciferase reporter assay

The HSPA8 promoter was inserted into the pGL3-basic vector, followed by co-transfection with 100 ng pRL-TK Renilla luciferase reporter into HepG2 and SK-HEP-1 cells using Lipofectamine 3000. After 48 h, the luciferase activity was tested using a luciferase reporter assay system (Promega, Madison, WI, USA) according to the manufacturer's instructions.

### 2.14 Chromatin immunoprecipitation (ChIP) assay

The ChIP Assay was performed using a ChIP Assay kit (#P2078, Beyotime) according to the manufacturer's instructions. Briefly, cells were treated with 1 % formaldehyde and collected for nuclear preparation and chromatin sonication into 200∼1000bp fragments. After centrifugation, the supernatant was incubated with 2 μg *anti*-p53 (#sc-126, Santa Cruz Biotechnology) at 4 °C overnight. Then, 60 μL Protein A/G agarose was added into the supernatant and incubated for 1 h at 4 °C. Enriched DNA fragments were purified for qPCR analysis.

### 2.15 DNA pull-down assay

Cells were collected and incubated with wild-type or mutant biotin-labelled HSPA8 promoter probes at 4 °C overnight, with gentle rotation. Next, cell lysate was incubated with streptavidin magnetic beads (#HY-K0208, MedChemExpress) for another 1 h at 4 °C. The enriched proteins were eluted for Western blot analysis.

### 2.16 Use of publicly available tool

Gene and protein levels in liver cancer and normal tissues were analysed using The University of ALabama at Birmingham CANcer Data Analysis Portal (UALCAN) [31], an integrated cancer data analysis platform containing The Cancer Genome Atlas (TCGA), Clinical Proteomic Tumour Analysis Consortium (CPTAC), and The Children Brain Tumor Tissue Consortium (CBTTC) datasets.

### 2.17 Statistical analysis

Data were presented as the mean ± standard deviation of at least three independent experiments carried out in triplicate. Comparisons between two groups were conducted using the unpaired Student's t-test. One-way or two-way analysis of variance (ANOVA) was used to analyse differences between multiple groups. Correlations among LACTB, p53, and HSPA8 were tested using Spearman's correlation coefficients. *P* < 0.05 was considered significant.

## 3 Results

### 3.1 LACTB acts as a tumour suppressor in liver cancer

**Fig. 1:**
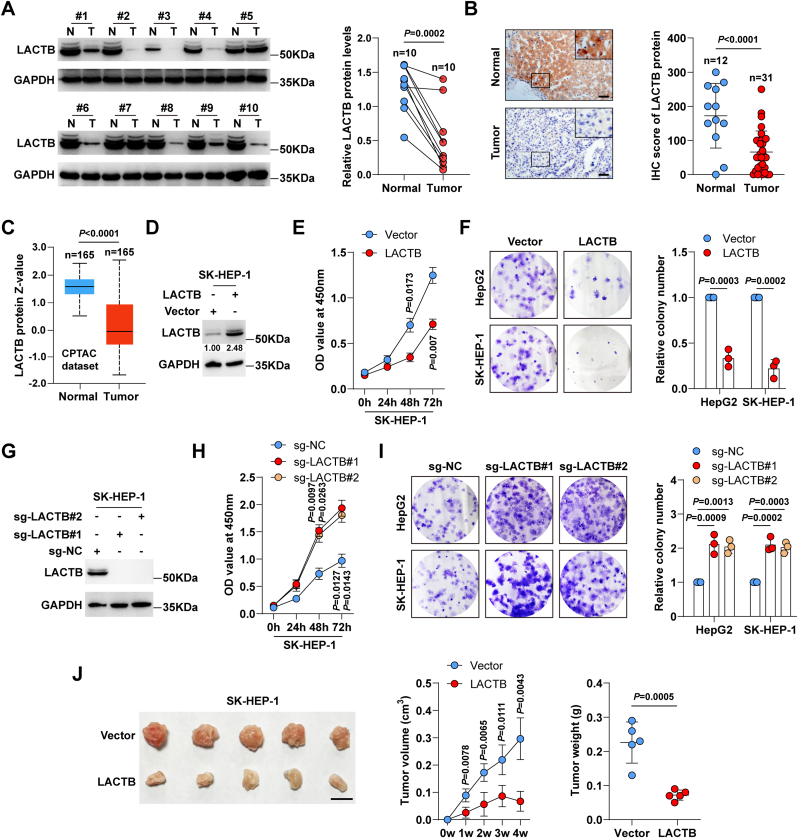
**LACTB inhibits liver cancer progression.** A. Western blot testing the protein levels of LACTB in ten paired liver cancer and normal tissues. B. IHC staining of LACTB in 12 normal and 31 liver cancer tissues. Scale bar = 50 μm. C. CPTAC database showing LACTB protein expression in normal and liver cancer tissues. D. Western blot verifying LACTB overexpression in SK-HEP-1 cells. E. CCK-8 assay testing cell viability in LACTB-expressing SK-HEP-1 cells. F. Colony formation assay testing the cloning ability of LACTB-expressing HepG2 and SK-HEP-1 cells. G. Western blot verifying LACTB knockout in SK-HEP-1 cells. H, I. CCK-8 and colony formation assays testing cell viability and cloning ability in LACTB^−/−^ cells, respectively. J. Xenograft tumour model testing the *in vivo* effects of LACTB overexpression on SK-HEP-1 cell growth. Scale bar = 1 cm. Paired Student's *t*-test was used for A, Student's *t*-test was used for B, F, J (right panel), two-way ANOVA with Sidak post-hoc test was used for E, H, J (middle panel), and one-way ANOVA with Dunnett post-hoc test was used for I.

We first tested LACTB expression in ten pairs of freshly frozen tissues (Fig. 1A) and found that LACTB protein was significantly downregulated in liver cancer tissues compared to that in the adjacent normal tissues. Furthermore, paraffin-embedded tissues were collected for IHC staining, and the results showed that LACTB was expressed at low levels in liver cancer cases (Fig. 1B). The Clinical Proteomic Tumour Analysis Consortium (CPTAC) database [31,32] also shows a decrease in LACTB protein expression in liver cancer (Fig. 1C). We then constructed LACTB-expressing SK-HEP-1 and HepG2 cell lines (Fig. 1D, Fig. S1A) and found that the ectopic expression of LACTB significantly inhibited cell viability (Fig. 1E, Fig. S1B) and colony formation (Fig. 1F). Furthermore, LACTB^−/−^ cell lines were also generated by using CRISPR/Cas9 gene editing technology (Fig. 1G, Fig. S1C). The results showed that cell viability (Fig. 1H, Fig. S1D) and colony formation (Fig. 1I) were notably enhanced after knockout of LACTB. To verify the role of LACTB *in vivo*, a xenograft tumour model was used. HepG2 cells were originally injected subcutaneously into mice, but no tumour formation was observed after one month. HepG2 cells were thus replaced with SK-HEP-1 cells, the results showed that overexpression of LACTB led to a decrease in tumour volume and weight (Fig. 1J), whereas LACTB knockout exerted the opposite effects (Fig. S1E). Overall, these data suggested that LACTB retarded liver cancer cell growth both *in vitro* and *in vivo*.

### 3.2 LACTB induces ferroptosis

**Fig. 2:**
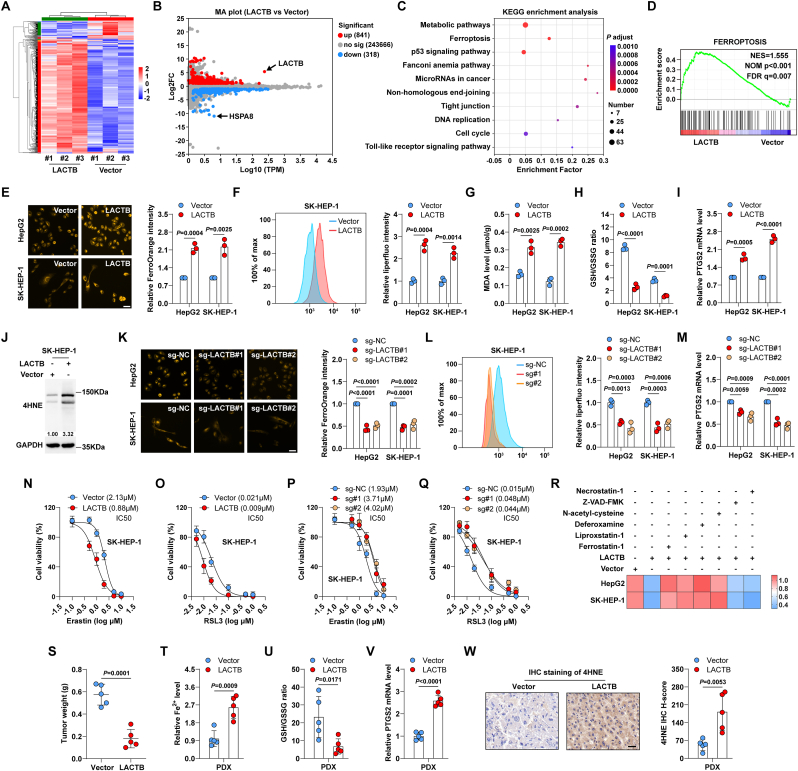
**LACTB as a driver of ferroptosis.** A. The heat map of differentially expressed genes in control and LACTB-expressing SK-HEP-1 cells. B. MA plot of the differentially expressed genes, red denotes upregulation, blue-green denotes downregulation, and gray denotes no difference. C. KEGG enrichment analysis showing signalling pathways for differential gene enrichment. D. GSEA showing the link between LACTB and ferroptosis. E. FerroOrange staining testing Fe^2+^ levels in LACTB-expressing HepG2 and SK-HEP-1 cells. Scale bar = 25 μm. F. Liperfluo staining testing lipid peroxidation in LACTB-expressing cells. G, H. Evaluation of the effects of LACTB overexpression on MDA levels and GSH/GSSG ratio. I. qRT-PCR analysis of PTGS2 mRNA expression in LACTB-expressing HepG2 and SK-HEP-1 cells. J. Western blot testing 4HNE protein expression in LACTB-expressing SK-HEP-1 cells. K-M. Detection of effects of LACTB knockout on Fe^2+^, lipid peroxidation, MDA, GSH/GSSG ratio, PTGS2 mRNA and 4HNE protein levels in cells. Scale bar = 25 μm. N, O. CCK-8 assay testing the viability of SK-HEP-1 cells expressing LACTB after treatment with erastin or RSL3. P, Q. CCK-8 assay testing the viability of LACTB^−/−^ cells treated with erastin or RSL3. R. CCK-8 assay testing the viability of cells expressing LACTB after treatment with the indicated chemicals. S. Tumour weight in vector and LACTB groups in the established PDX models. T-V. Detection of Fe2+, GSH/GSSG ratio and PTGS2 mRNA levels in vector and LACTB groups. W. IHC staining of PDX tumour tissues using anti-4HNE antibody. Scale bar = 50 μm. Student's *t*-test was used for *E*-I, *S*–W, and one-way ANOVA with Dunnett post-hoc test was used for K-M. (For interpretation of the references to color in this figure legend, the reader is referred to the Web version of this article.)

To understand the function of LACTB, we performed RNA sequencing of LACTB-expressing SK-HEP-1 cells (Fig. 2A). A group of genes (841 upregulated and 318 downregulated) was associated with LACTB (Fig. 2B), involving key pathways, such as metabolic pathways, ferroptosis, and p53 signalling pathways (Fig. 2C). Importantly, the Gene Set Enrichment Analysis (GSEA) showed that high LACTB was significantly positively correlated with ferroptosis (Fig. 2D). Therefore, we inferred that LACTB represses liver cancer via ferroptosis. As expected, LACTB overexpression markedly increased Fe^2+^ (Fig. 2E), lipid peroxidation (Fig. 2F, Fig. S2A), and MDA (Fig. 2G) levels but reduced GSH levels (Fig. 2H). Moreover, the ferroptosis markers PTGS2 (Fig. 2I) and 4HNE (Fig. 2J, Fig. S2B) were notably increased after LACTB overexpression. Conversely, the levels of Fe^2+^, lipid peroxidation, MDA, PTGS2, and 4HNE were dramatically decreased, while the GSH content was increased in LACTB^−/−^ cells compared to control cells (Fig. 2K-M, Figs. S2C–F). Moreover, a mouse model confirmed the ferroptosis-promoting effects of LACTB *in vivo* (Figs. S2G and H). Enforced expression of LACTB endowed HepG2 and SK-HEP-1 cells with greater sensitivity to the ferroptosis inducers erastin and RSL3 (Fig. 2N, O, Figs. S2I and J), whereas LACTB knockout resulted in the opposite effects (Fig. 2P, Q, Fig. S2K-M). The reduced cell viability caused by LACTB was effectively rescued by ferrostatin-1/liproxstatin-1 (a ferroptosis inhibitor), deferoxamine (an iron chelator), and *N*-acetyl-cysteine (an ROS scavenger), but not by *Z*-VAD-FMK (a pan-caspase inhibitor) or necrostatin-1 (a necroptosis inhibitor) (Fig. 2R). In addition, the proportion of apoptotic cells slightly increased after LACTB overexpression (Fig. S2N).

To make the results more clinically relevant, we established a PDX model, followed by intratumoural injection of an adeno-associated virus-expressing vector or LACTB. The results showed that LACTB overexpression significantly retarded tumour growth (Fig. 2S), accompanied by the elevation of Fe^2+^, PTGS2, and 4HNE and the downregulation of GSH (Fig. 2T-W).

Collectively, these results indicate that LACTB is a potential driver of ferroptosis in liver cancer.

### 3.3 LACTB triggers ferroptosis via inhibiting HSPA8

**Fig. 3:**
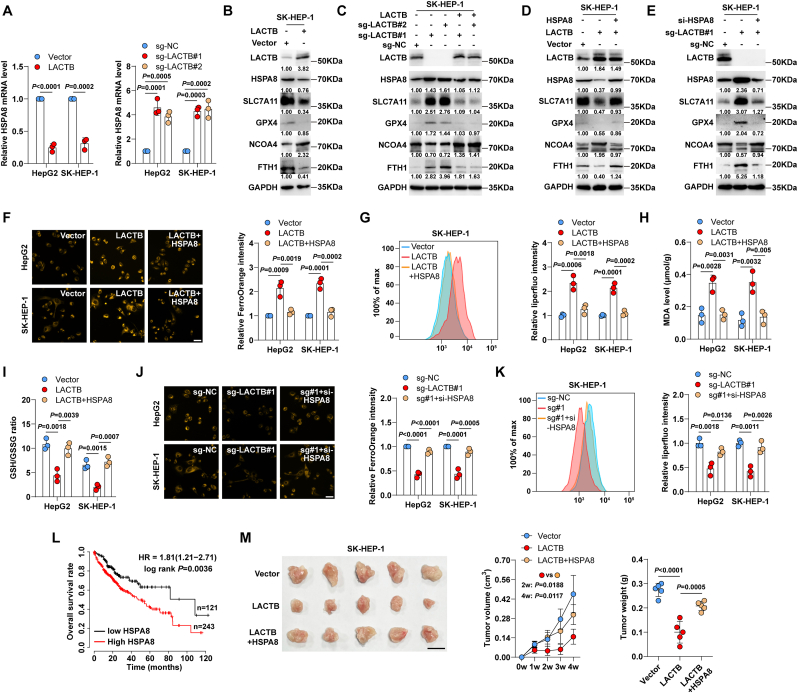
**HSPA8 is a target of LACTB.** A. qRT-PCR testing the effects of LACTB overexpression and knockout on HSPA8 mRNA levels. B. Western blot testing the indicated protein levels after LACTB overexpression in SK-HEP-1 cells. C. Western blot testing the indicated protein levels in LACTB^−/−^ cells reexpressing LACTB. D. Western blot testing the indicated protein levels in LACTB-expressing cells transfected with HSPA8-expressing plasmid. E. Western blot testing the indicated protein levels in LACTB^−/−^ cells transfected with si-HSPA8. F–I. Detection of effects of LACTB overexpression or in combination with HSPA8 overexpression on Fe^2+^, lipid peroxidation, MDA levels and GSH/GSSG ratio in cells. Scale bar = 25 μm. J,K. Detection of effects of LACTB knockout or in combination with HSPA8 silencing on Fe^2+^ and lipid peroxidation levels in HepG2 and SK-HEP-1 cells. Scale bar = 25 μm. L. Kaplan-Meier plotter database showing the overall survival rate of liver cancer patients with high or low HSPA8 expression. M. Xenograft tumour model testing the *in vivo* effects of LACTB or LACTB + HSPA8 on SK-HEP-1 cell growth. Scale bar = 1 cm. Student's *t*-test was used for A (left panel), one-way ANOVA with Tukey post-hoc test was used for A (right panel), F–K, M (right panel), and two-way ANOVA with Tukey post-hoc test was used for M (middle panel).

As shown in Fig. 2B, HSPA8 mRNA abundance was most significantly reduced after LACTB overexpression. Given that HSPA8 blocks ferroptosis by promoting SLC7A11/GSH/GPX4 signalling and inhibiting NCOA4-mediated ferritinophagy [33,34], we speculated that HSPA8 may be the downstream target of LACTB. As expected, HSPA8 mRNA expression was decreased and increased by LACTB overexpression and knockout, respectively (Fig. 3A). Consistently, the protein levels of HSPA8, SLC7A11, GPX4, and FTH1 were reduced, whereas those of NCOA4 were increased in LACTB-expressing HepG2 and SK-HEP-1 cells (Fig. 3B, Fig. S3A). Knockout of LACTB resulted in the opposite effects; however, re-expression of LACTB in LACTB^−/−^ cells abolished these effects (Fig. 3C, Fig. S3B). Furthermore, the decrease in SLC7A11, GPX4, and FTH1 levels and the increase in NCOA4 levels caused by LACTB overexpression were blocked by HSPA8 overexpression (Fig. 3D, Fig. S3C). Likewise, the increased SLC7A11, GPX4, and FTH1 and the decreased NCOA4 induced by LACTB knockout were also counteracted after silencing HSPA8 (Fig. 3E, Fig. S3D). Moreover, the ferroptotic phenotypes caused by LACTB overexpression and knockout were significantly abrogated by HSPA8 overexpression and knockdown (Fig. 3F–K, Figs. S3E–H), respectively. TCGA and CPTAC databases showed that HSPA8 mRNA and protein levels were notably increased in liver cancer tissues compared to those in normal tissues (Fig. S3I). High HSPA8 expression was linked to a dismal overall survival (Fig. 3L), as illustrated by the Kaplan-Meier plotter database [35]. Importantly, the reduced tumour size (Fig. 3M) and increased Fe^2+^, PTGS2, and 4HNE levels (Fig. S3J) caused by LACTB overexpression were significantly rescued by HSPA8 overexpression. Taken together, these data suggested that HSPA8 was responsible for the regulatory effects of LACTB on ferroptosis in liver cancer cells.

Inhibition of HSPA8 by LACTB requires wild-type p53.

**Fig. 4:**
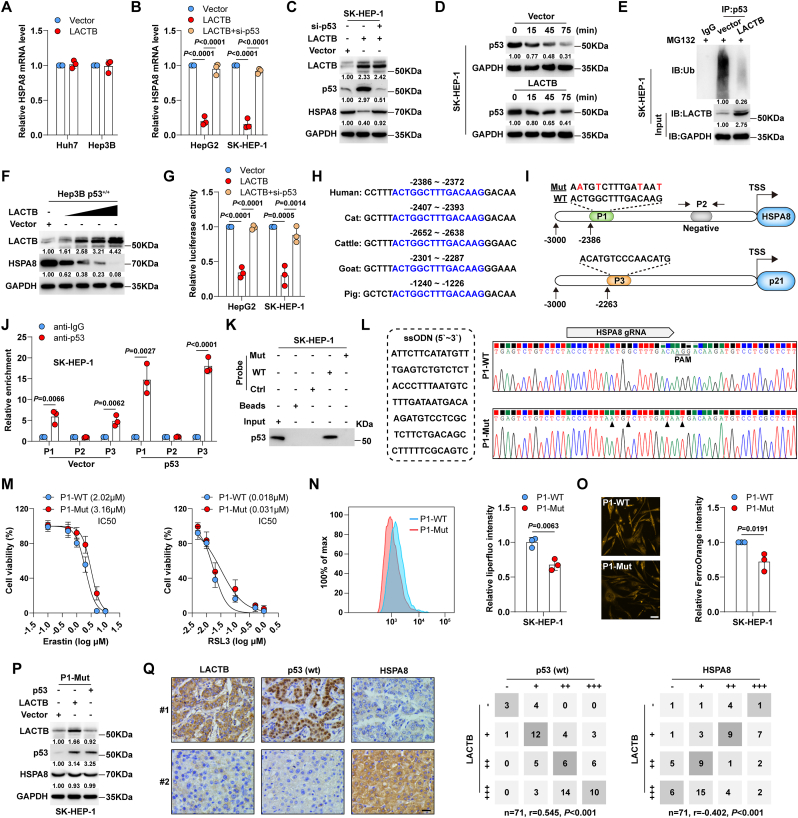
**LACTB drives ferroptosis via regulating the p53/HSPA8 axis.** A. qRT-PCR testing HSPA8 mRNA levels in LACTB-expressing Huh7 and Hep3B cells. B, C. qRT-PCR and Western blot testing HSPA8 mRNA and protein levels in LACTB-expressing cells transfected with si-p53. D. Western blot testing p53 protein expression in LACTB-expressing SK-HEP-1 cells treated with 100 μg/mL cycloheximide for the indicated time. E. Western blot testing p53 ubiquitination levels in LACTB-expressing SK-HEP-1 cells. F. Western blot testing HSPA8 protein expression in LACTB-expressing p53^+/+^ Hep3B cells. G. Luciferase reporter assay testing HSPA8 promoter activity in LACTB-expressing cells transfected with si-p53. H. p53 binding sites on HSPA8 promoter among different species. I. The schema showing the wild-type or mutant p53 binding motifs on HSPA8 or p21 promoter, and primer design positions for ChIP assay. J. ChIP assay testing the binding of p53 on the indicated regions of HSPA8 promoter. K. DNA pull-down assay using wild-type or mutant HSPA8 promoter probe, followed by Western blot analysis of p53 protein expression. L. Generation of SK-HEP-1 cells with endogenous mutation of p53 binding site in HSPA8 promoter using CRISPR/Cas9 gene editing technology with the indicated ssODN and gRNA. M. CCK-8 testing the viability of wild-type or mutant SK-HEP-1 cells treated with erastin and RSL3. N, O. Liperfluo and FerroOrange staining testing lipid peroxidation and Fe^2+^ levels in wild-type or mutant SK-HEP-1 cells. Scale bar = 25 μm. P. Western blot testing HSPA8 protein levels in wild-type or mutant SK-HEP-1 cells with LACTB or p53 overexpression. Q. IHC staining of LACTB, wild-type (wt) p53 and HSPA8 in liver cancer tissue microarray, followed by analysis of their expression correlations. Scale bar = 50 μm. One-way ANOVA with Tukey post-hoc test was used for B and G. Student's *t*-test was used for J, N, O.

Intriguingly, LACTB overexpression did not affect Fe^2+^ (Fig. S4A), lipid peroxidation (Fig. S4B), PTGS2 (Fig. S4C), or erastin sensitivity (Fig. S4D) in p53-mutant Huh7 and p53-null Hep3B cells. Our previous study showed that LACTB inhibits colorectal cancer progression in a wild-type p53-dependent manner [24]; LACTB can directly bind to p53 protein to inhibit p53 ubiquitination degradation by preventing MDM2 from interacting with p53 [24]. Hence, we assumed that LACTB expression was the same in liver cancer. HSPA8 mRNA (Fig. 4A) and protein (Figs. S4E and F) levels were unaltered in Huh7 and Hep3B cells expressing LACTB, and knockdown of endogenous p53 had no effect on HSPA8 expression in Huh7 cells (Fig. S4G). However, the decrease in HSPA8 caused by LACTB overexpression in HepG2 and SK-HEP-1 cells harbouring wild-type p53 was significantly elevated after p53 silencing (Fig. 4B, C, Fig. S4H), suggesting that wild-type p53 is required for LACTB-mediated regulation of HSPA8. The half-life of p53 was significantly prolonged after LACTB overexpression in HepG2 and SK-HEP-1 cells (Fig. 4D, Fig. S4I), accompanied by a reduction in p53 ubiquitination (Fig. 4E, Fig. S4J), consistent with our previous findings [24]. Moreover, we introduced p53 into p53-null Hep3B cells (Fig. S4K) and found that LACTB reduced HSPA8 expression in a dose-dependent manner (Fig. 4F).

To understand how HSPA8 mRNA is regulated by the LACTB/p53 axis, we tested the promoter activity of HSPA8. The results of the luciferase reporter assay showed that LACTB overexpression significantly inhibited HSPA8 promoter activity, whereas p53 knockdown blocked this effect (Fig. 4G). By analysing the sequence of the HSPA8 promoter, a highly conserved p53 binding motif was found at −2386∼-2372 (Fig. 4H). We then designed a series of primers for the chromatin immunoprecipitation (ChIP) assay (Fig. 4I), which showed that p53 directly binds to the HSPA8 promoter (p21 is a well-known target of p53 and was used as a positive control) (Fig. 4J, Fig. S4L). The DNA pull-down assay showed that p53 protein was significantly enriched by the wild-type HSPA8 promoter probe, but not by the mutant probe (mutation of the “CWWG” core) (Fig. 4K, Fig. S4M). P53 overexpression markedly repressed the transcription of the wild-type HSPA8 promoter but did not affect that of the mutated promoter (Figs. S4N and O). Furthermore, we employed CRISPR/Cas9 genome editing with a single-stranded oligodeoxynucleotide (ssODN) and generated SK-HEP-1 cells with an endogenous mutation of p53 binding site in the HSPA8 promoter (mutation of four nucleotides from ACTGGCTTTGACAAG to AATGTCTTTGATAAT) (Fig. 4L). Compared with wild-type cells, mutant cells were less sensitive to the ferroptosis inducers erastin and RSL3 (Fig. 4M) and had lower lipid peroxidation (Fig. 4N) and Fe^2+^ levels (Fig. 4O). As shown in Fig. S4P, p53 did not bind to the HSPA8 promoter in mutant cells, and the overexpression of p53 or LACTB had no effect on HSPA8 expression (Fig. S4Q, Fig. 4P). Similarly, the ferroptosis-promoting effect of LACTB was substantially attenuated in the mutant cells (Fig. S4R). Finally, we detected the expression of LACTB, wild-type p53, and HSPA8 using a tissue microarray containing 71 liver cancer cases. The results showed that LACTB was positively and negatively correlated with wild-type p53 (r = 0.545) and HSPA8 (r = −0.402), respectively (Fig. 4Q).

Collectively, these findings demonstrate that LACTB dictates ferroptosis by regulating the p53/HSPA8 axis in liver cancer.

### 3.4 LACTB enhances the response of liver cancer to lenvatinib

**Fig. 5:**
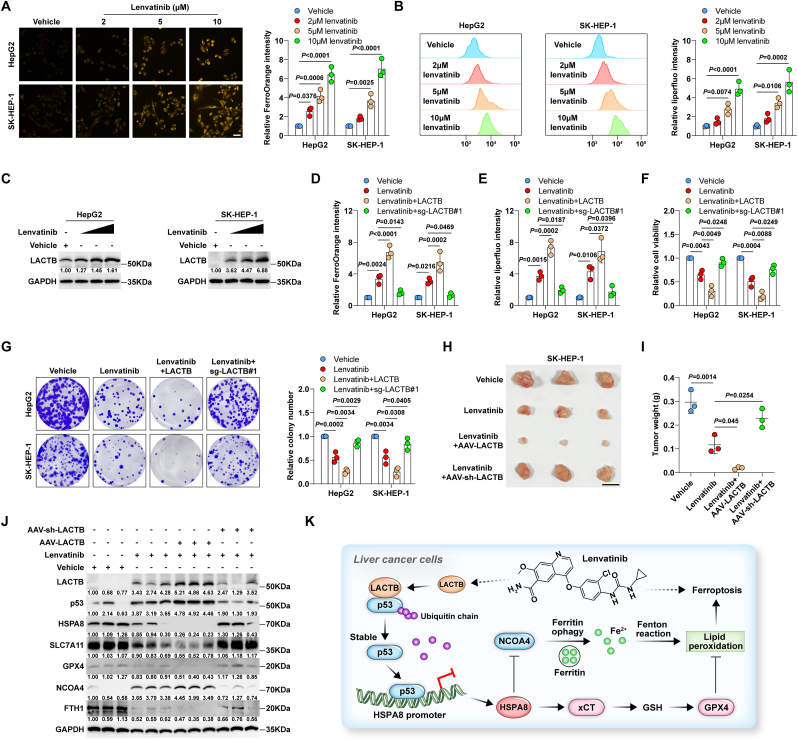
**LACTB potentiates the response of liver cancer to lenvatinib.** A, B. FerroOrange and liperfluo staining testing Fe^2+^ and lipid peroxidation levels in HepG2 and SK-HEP-1 cells treated with lenvatinib, respectively. Scale bar = 25 μm. C. Western blot testing LACTB protein expression in HepG2 and SK-HEP-1 cells treated with lenvatinib. D-G. FerroOrange, liperfluo staining, CCK-8 and colony formation assays respectively testing Fe^2+^, lipid peroxidation levels, viability and cloning ability in lenvatinib-treated cells with LACTB overexpression or knockout. H, I. Xenograft tumour model testing the *in vivo* effects of LACTB overexpression or knockout on the anti-tumour effect of lenvatinib. Scale bar = 1 cm. J. Western blot testing the indicated protein expression in the indicated groups. K. The proposed model showing that LACTB induced by lenvatinib promotes ferroptosis by increasing p53 protein stability and inhibiting HSPA8-mediated *anti*-ferroptosis pathway. One-way ANOVA with Tukey post-hoc test was used for A, B, D-G, I.

Recent evidence suggests that the tyrosine kinase inhibitor lenvatinib is a potential ferroptosis inducer [36,37], and the above data demonstrate that LACTB induces ferroptosis. Thus, we wondered whether there is a link between LACTB and lenvatinib. As shown in Fig. 5A and B, lenvatinib increased Fe^2+^ and lipid peroxidation levels in a dose-dependent manner, implying that lenvatinib induces ferroptosis. Notably, LACTB protein levels increased after lenvatinib treatment of HepG2 and SK-HEP-1 cells (Fig. 5C). The increase in Fe^2+^ and lipid peroxidation levels and the decrease in cell viability and colony formation caused by lenvatinib were more pronounced with LACTB overexpression (Fig. 5D–G), while LACTB knockout blocked these effects (Fig. 5D–G). Next, we assessed the *in vivo* effects of LACTB on lenvatinib using a xenograft tumour model. When the volume of subcutaneous tumour reached about 0.05–0.1 cm^3^, mice were treated with lenvatinib and intratumorally injected with adeno-associated virus expressing LACTB or sh-LACTB. The results showed that overexpression and knockdown significantly enhanced and attenuated the anti-tumour efficacy of lenvatinib, respectively (Fig. 5H and I). Furthermore, LACTB, p53, NCOA4, Fe^2+^, PTGS2, and 4HNE were increased (Fig. 5J, Figs. S5A–C), while HSPA8, SLC7A11, GPX4, and FTH1 were decreased in the lenvatinib group compared to those in the vehicle group (Fig. 5J); these phenomena became pronounced and attenuated in the lenvatinib + LACTB and lenvatinib + sh-LACTB groups, respectively (Fig. 5J, Figs. S5A–C). These data indicate that LACTB is a downstream effector of lenvatinib and that the antitumour efficacy of lenvatinib is potentiated by LACTB in liver cancer.

## 4 Discussion

A key challenge in cancer research is the effective killing of tumour cells while keeping healthy cells intact. Tumour cells are often defective in their death mechanisms, which is a major reason for treatment failure in clinical settings [38]. To meet their growth needs, tumour cells have a higher demand for iron compared to non-tumour cells, which makes tumour cells more susceptible to iron-catalysed death, namely ferroptosis [39]. Therefore, the appropriate manipulation of ferroptosis is considered a promising avenue to combat cancer and improve therapeutic efficacy [40], making it particularly important to elucidate the regulatory networks of ferroptosis. In the present study, we identified a previously unknown intrinsic driver of ferroptosis in liver cancer. LACTB stabilises the p53 protein to inhibit HSPA8 transcription, resulting in the activation of NCOA4-mediated ferritinophagy and the inhibition of SLC7A11/GSH/GPX4 signalling, thereby triggering ferroptosis and repressing liver tumourigenesis (Fig. 5K). Notably, a recent study showed that extensive succinylation of LACTB K284, induced by OXCT1, inhibits LACTB activity and promotes cancer progression [41], implying a strong link between LACTB and liver cancer. The above study and our data demonstrate the downregulation of LACTB in liver cancer tissues. Whether this is directly related to succinylation needs to be investigated in depth; if not, there may be other post-translational modifications involved in LACTB protein turnover, such as ubiquitination. Taken together, our findings provide robust evidence that LACTB is an intrinsic inducer of ferroptosis, and that restoration of LACTB may be a potentially effective therapeutic approach against liver cancer.

HSPA8, a member of the heat shock protein 70 family, is a constitutively expressed molecular chaperone [42]. HSPA8 cooperates with co-chaperones to regulate its target proteins, thereby affecting various cellular functions and biological processes [43,44]. HSPA8 is frequently overexpressed in human cancers and promotes cancer development and progression in a context-dependent manner [45,46]. Here, we found that HSPA8 is significantly upregulated in liver cancer, associated with poor prognosis. Overexpression of HSPA8 notably abolishes the antitumour effect of LACTB, suggesting that HSPA8 is an oncogene in liver cancer. Several studies have shown that HSPA8 inhibits ferroptosis by promoting SLC7A11/GSH/GPX4 signalling and inhibiting NCOA4-mediated ferritinophagy [33,34], We verified the effects of HSPA8 in liver cancer and found that LACTB promotes ferroptosis by regulating HSPA8-mediated ferroptosis-related signalling. Future research is needed to investigate how HSPA8 affects the expression of SLC7A11 and NCOA4 to regulate ferroptosis, which is likely related to its ability to shuttle proteins between the cytoplasm, nucleus, and organelles, such as in a cellular recycling program called chaperone-mediated autophagy (CMA) [44,47].

Lenvatinib is a small-molecule inhibitor used to treat advanced liver cancer with more focused targets of action, stronger inhibition, and fewer side effects than those by sorafenib [3]. However, the overall response rate to lenvatinib is only 24.1 %, highlighting the need to identify combination therapies that enhance its clinical benefits [3]. In this study, we confirmed results of recent studies reporting that lenvatinib induces ferroptosis [36,37,48]. Unexpectedly, LACTB was found to be a target of lenvatinib. Lenvatinib increases LACTB expression in a dose-dependent manner. Moreover, adeno-associated virus-mediated overexpression of LACTB prominently enhanced the tumour-inhibiting effect of lenvatinib in a mouse model, suggesting that the combination of lenvatinib and LACTB may be helpful for the treatment of patients with liver cancer for whom lenvatinib alone has low efficacy. Follow-up studies are needed to address how lenvatinib elevates LACTB protein levels and whether LACTB is a direct or indirect target of lenvatinib.

In conclusion, our data shed light on the importance of LACTB in driving ferroptosis. Targeting the newly identified LACTB/p53/HSPA8 axis may be an effective therapeutic strategy in patients with liver cancer.

## Funding

This study was supported by grants from the National Natural Science Foundation of China (82,303,440, 82,173,207), Natural Science Basic Research Program of Shaanxi Province (2022JQ-825, 2022JQ-863), the Key Research and Development Program of Shaanxi Province of China (2021SF-381), Shaanxi Provincial Health Research Fund (2022E022), Project funded by China Postdoctoral Science Foundation (2021M700108, 2022T150520), Fundamental Research Funds for the Central Universities (xzy012021063) and Free Exploration and Innovation Fund (2020 YJ (ZYTS)546–12).

## Ethics approval

The use of clinical specimens and animal study were approved by the Biomedical Ethics Committee of Xi ‘an Jiaotong University Health Science Center (Approval No: 2022–970).

## CRediT authorship contribution statement

**Kaixuan Zeng:** Writing – original draft, Software, Methodology, Investigation, Funding acquisition, Formal analysis, Data curation. **Na Huang:** Methodology, Investigation, Formal analysis, Data curation. **Nanbin Liu:** Software, Resources, Methodology. **Xi Deng:** Validation, Software, Methodology. **Yanhua Mu:** Visualization, Resources. **Xurui Zhang:** Resources, Methodology. **Jian Zhang:** Visualization, Validation. **Chongyu Zhang:** Methodology. **Yong Li:** Visualization. **Zongfang Li:** Writing – review & editing, Writing – original draft, Supervision, Project administration, Funding acquisition, Conceptualization.

## Declaration of competing interest

The authors declare that they have no known competing financial interests or personal relationships that could have appeared to influence the work reported in this paper.

## Data Availability

Data will be made available on request.
