# Supplementary material for: LACTB suppresses liver cancer progression through regulation of ferroptosis
Source: Redox Biol. 2024 Jul 18;75:103270. doi: 10.1016/j.redox.2024.103270 (PMC11321384; doi:10.1016/j.redox.2024.103270)
Supplement: Multimedia component 1 [file mmc1.pdf]

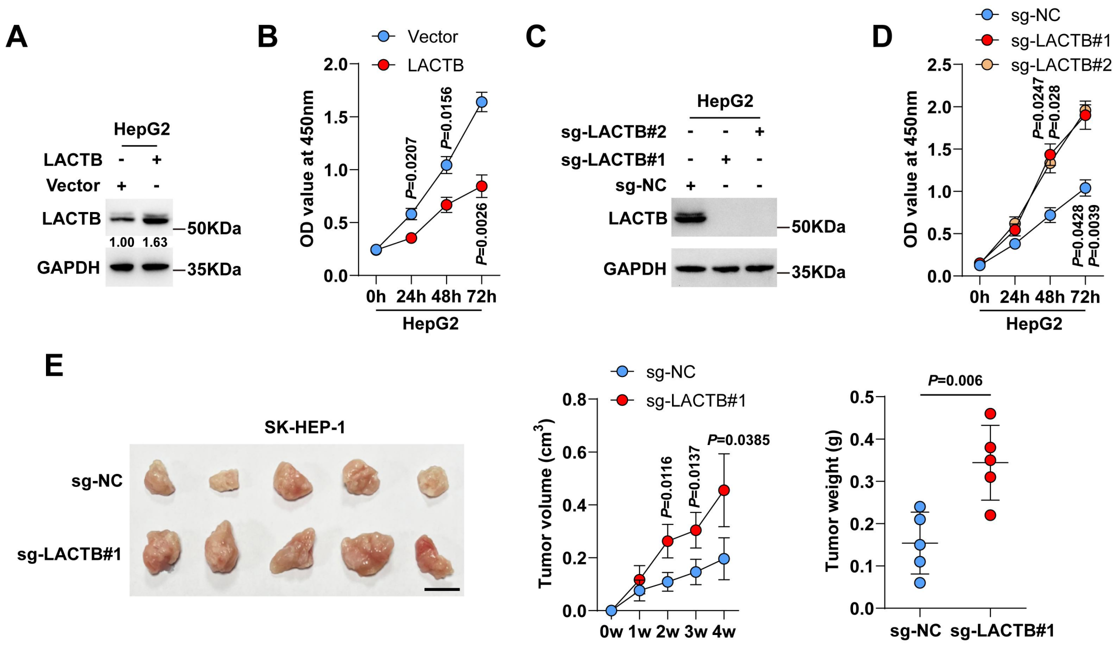

Figure S1. LACTB inhibits liver cancer. A. Western blot verifying LACTB overexpression in HepG2 cells. B. CCK-8 assay testing cell viability in LACTB-expressing HepG2 cells. C. Western blot verifying LACTB knockout in SK-HEP-1 cells. D. CCK-8 testing cell viability in $LACTB^{-/-}$ HepG2 cells. E. Xenograft tumor model testing the *in vivo* effects of LACTB knockout on SK-HEP-1 cell growth. Scale bar=1cm. Two-way ANOVA with Sidak post-hoc test was used for B, D, E (middle panel), Student's t test was used for E (right panel).

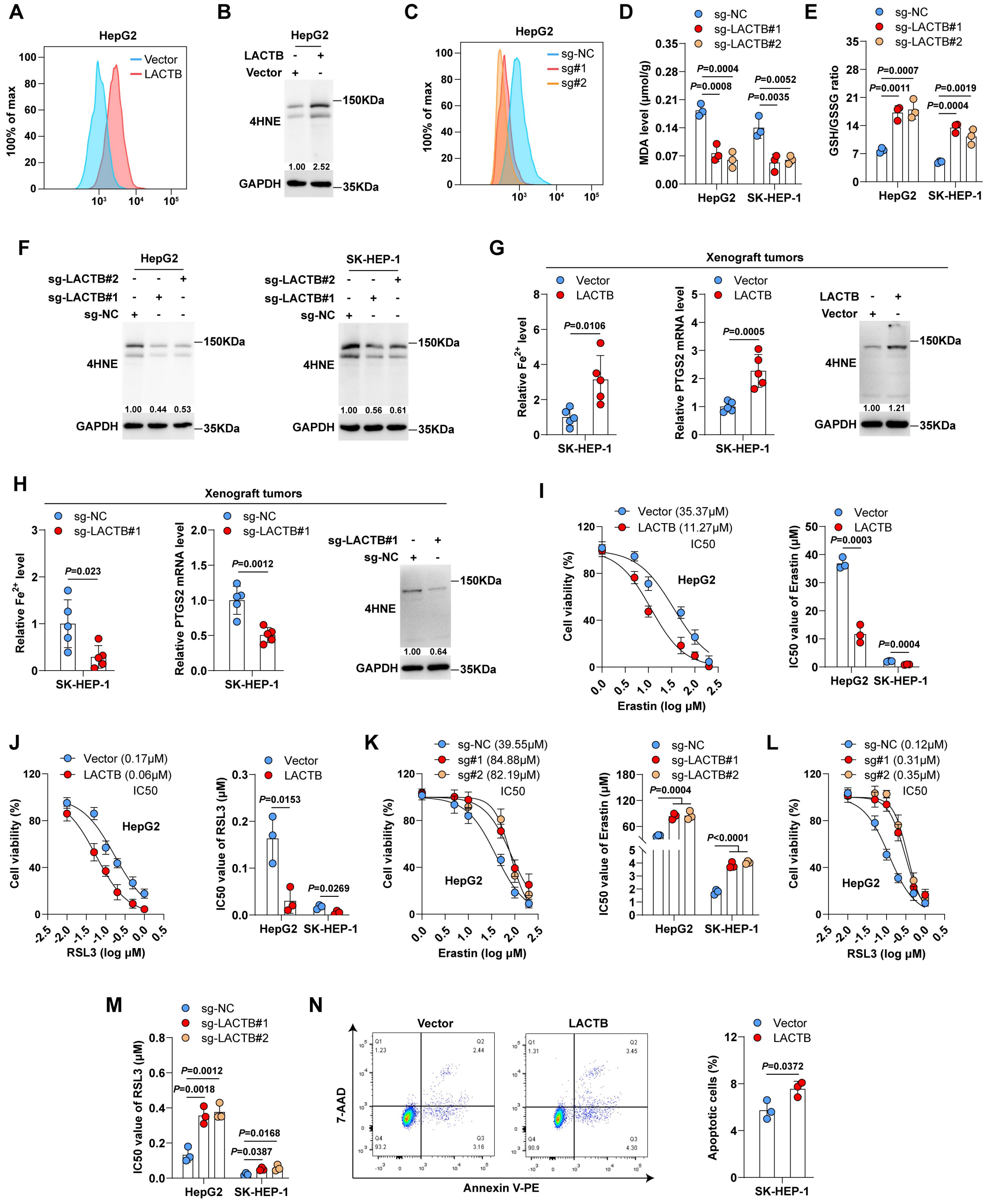

Figure S2. LACTB induces ferroptosis. A. Liperfluo staining testing lipid peroxidation in LACTB-expressing HepG2 cells. B. Western blot testing 4HNE protein expression in LACTB-expressing HepG2 cells. C. Liperfluo staining testing lipid peroxidation in LACTB$^{-/-}$ HepG2 cells. D, E. Evaluation of the effects of LACTB knockout on MDA levels and GSH/GSSG ratio. F. Western blot testing

4HNE protein expression in $LACTB^{-/-}$ HepG2 and SK-HEP-1 cells. G, H. Detection of Fe^{2+}, PTGS2 and 4HNE levels in tumor tissues with LACTB overexpression or knockout. I-M. CCK-8 assay testing the viability of cells with LACTB overexpression or knockout after treatment with erastin or RSL3. N. Flow cytometry detecting the effect of LACTB overexpression on cell apoptosis. One-way ANOVA with Dunnett post-hoc test was used for D, E, K, M. Student's t test was used for G-J, N.

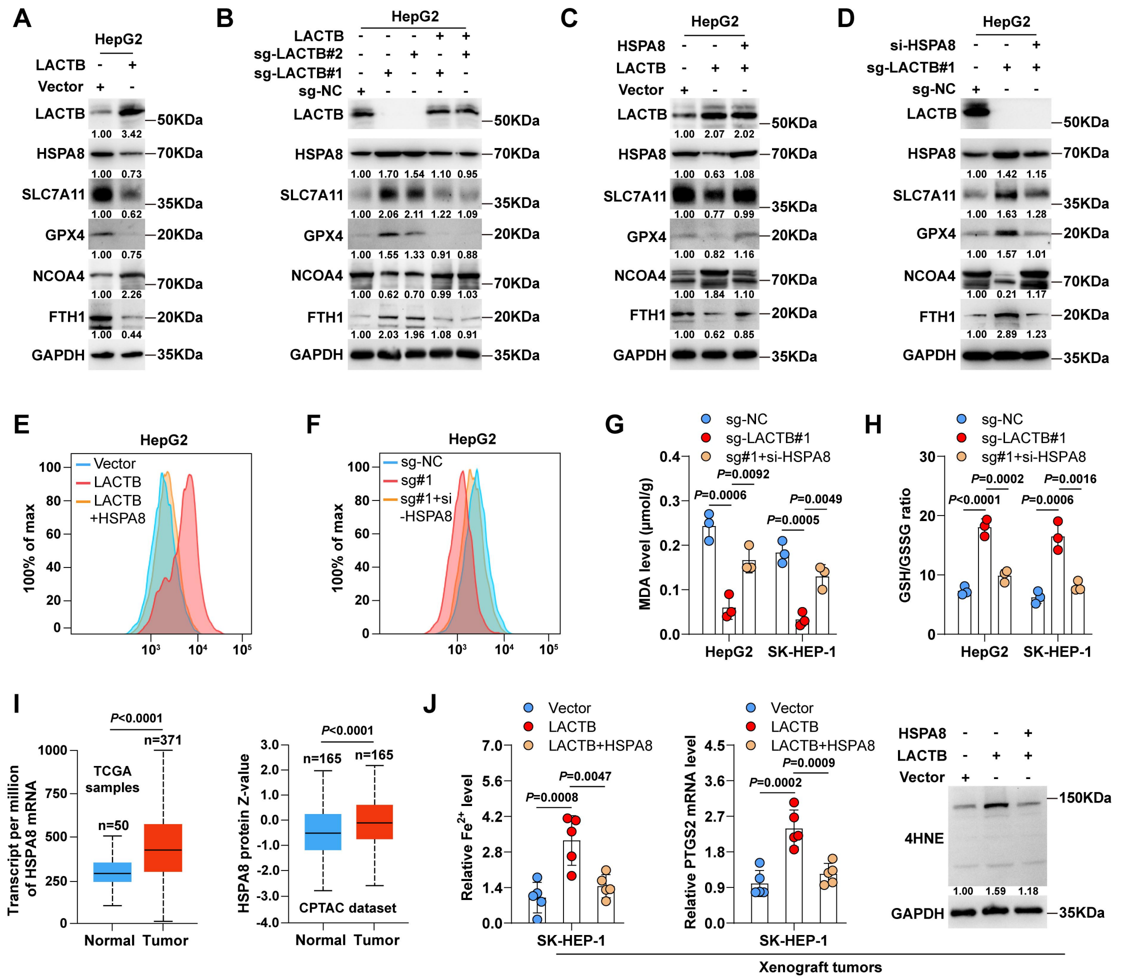

Figure S3. LACTB regulates HSPA8. A. Western blot testing the indicated protein levels after LACTB overexpression in HepG2 cells. B. Western blot testing the indicated protein levels in LACTB$^{-/-}$ cells reexpressing LACTB. C. Western blot testing the indicated protein levels in LACTB-expressing cells transfected with HSPA8-expressing plasmid. D. Western blot testing the indicated protein levels in LACTB$^{-/-}$ cells transfected with si-HSPA8. E. Liperfluo staining testing lipid peroxidation in LACTB-expressing cells transfected with HSPA8-expressing plasmid. F. Liperfluo staining testing lipid peroxidation in LACTB$^{-/-}$ cells transfected with si-HSPA8. G, H. Detection of MDA levels and GSH/GSSG ratio in LACTB$^{-/-}$ cells transfected with si-HSPA8. I. TCGA database showing the expression of HSPA8

mRNA in 50 normal and 371 liver cancer tissues (left), CPTAC database showing HSPA8 protein expression in normal and liver cancer tissues (right). J. Detection of Fe^{2+}, PTGS2 mRNA and 4HNE levels in tumor tissues expressing LACTB or LACTB+HSPA8. One-way ANOVA with Tukey post-hoc test was used for G, H, J.

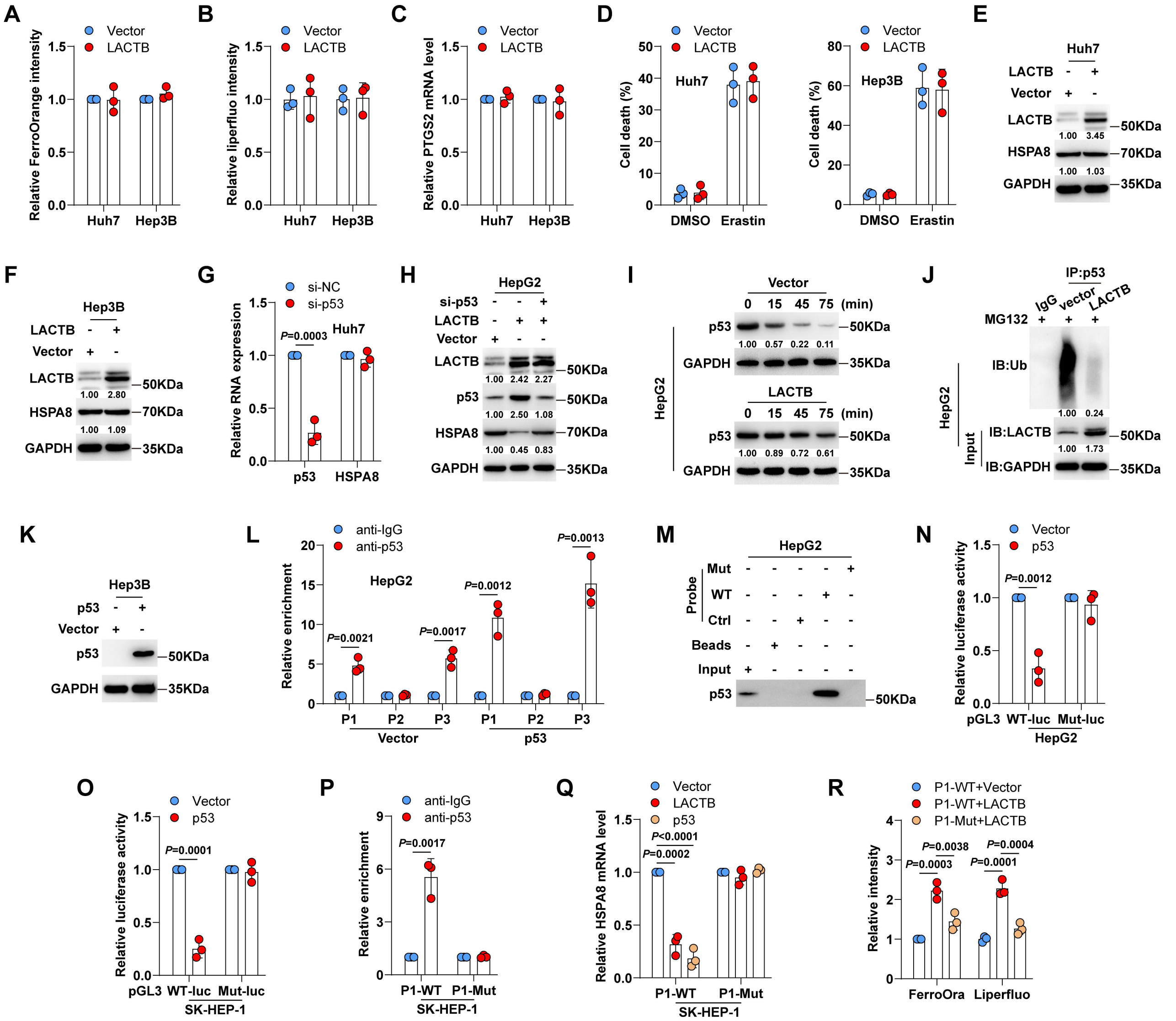

Figure S4. LACTB regulates HSPA8 via p53. A. FerroOrange and liperfluo staining testing Fe^{2+} and lipid peroxidation levels in LACTB-expressing Huh7 and Hep3B cells, respectively. C. qRT-PCR analysis of PTGS2 mRNA levels in LACTB-expressing

Huh7 and Hep3B cells. D. Propidium iodide staining testing the death of LACTB-expressing Huh7 and Hep3B cells treated with erastin. E, F. Western blot testing HSPA8 protein levels in LACTB-expressing Huh7 and Hep3B cells. G. qRT-PCR analysis of HSPA8 mRNA levels in Huh7 cells transfected with p53 siRNA. H. Western blot testing HSPA8 protein levels in LACTB-expressing HepG2 cells transfected with p53 siRNA. I. Western blot testing p53 protein expression in LACTB-expressing HepG2 cells treated with 100μg/mL cycloheximide for the indicated time. J. Western blot testing p53 ubiquitination levels in LACTB-expressing HepG2 cells. K. Western blot verifying the overexpression efficiency of p53 in Hep3B cells. L. ChIP assay testing the binding of p53 on the indicated regions of HSPA8 promoter. M. DNA pull-down assay using wild-type or mutant HSPA8 promoter probe, followed by western blot analysis of p53 protein expression. N, O. Luciferase reporter assay testing the effects of p53 overexpression on HSPA8 promoter activity. P. ChIP assay testing the binding of p53 on HSPA8 promoter in wild-type or mutant SK-HEP-1 cells. Q. qRT-PCR testing HSPA8 mRNA levels in wild-type or mutant SK-HEP-1 cells with LACTB or p53 overexpression, respectively. R. FerroOrange and liperfluo staining testing Fe^{2+} and lipid peroxidation levels in wild-type or mutant SK-HEP-1 cells with LACTB overexpression. Student's t test was used for G, L, N-P. One-way ANOVA with Tukey post-hoc test was used for Q and R.

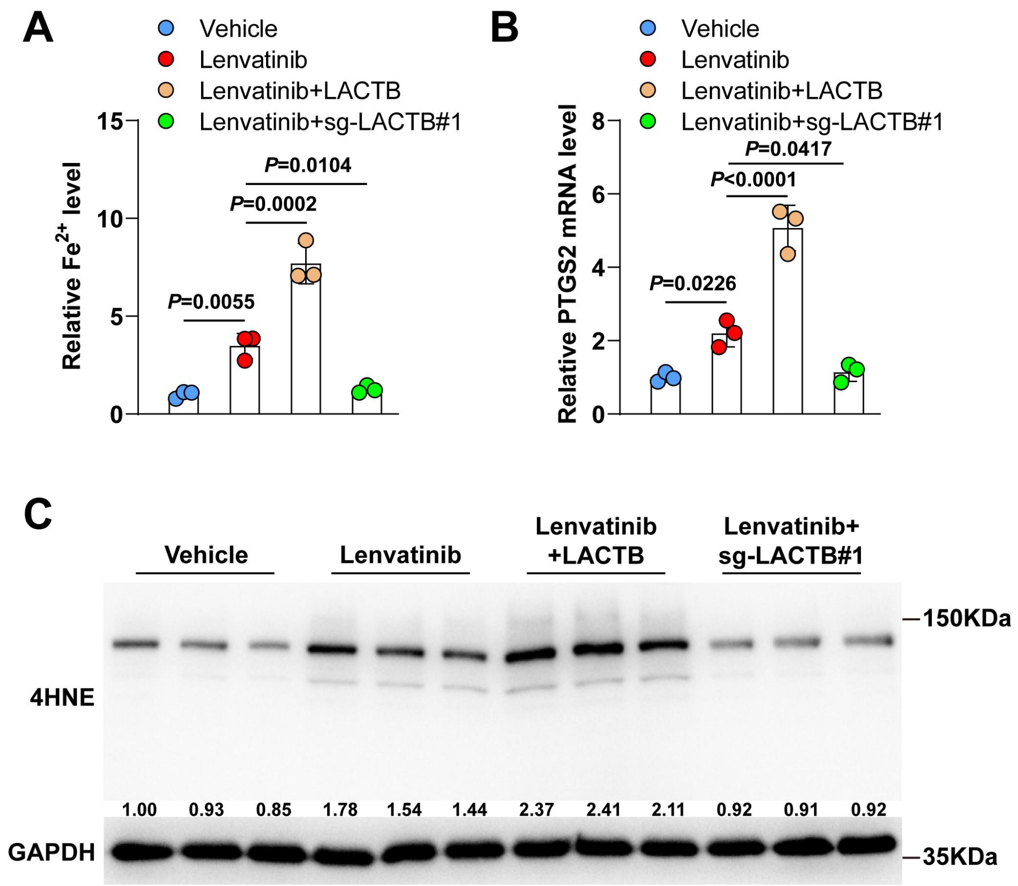

Figure S5. LACTB affects the pro-ferroptosis effect of lenvatinib. A. Detection of Fe^{2+} levels in the indicated four groups. B, C. qRT-PCR and western blot analysis of PTGS2 mRNA and 4HNE protein levels in the indicated four groups, respectively. One-way ANOVA with Tukey post-hoc test was used for A, B.
